# Influenza Vaccination Among Adults 65 Years or Older: A 2009–2010 Community Health Survey in the Honam Region of Korea

**DOI:** 10.3390/ijerph8114197

**Published:** 2011-11-08

**Authors:** So Yeon Ryu, So Hui Kim, Hyung Su Park, Jong Park

**Affiliations:** Department of Preventive Medicine, Chosun University Medical School, 375 Seosuk-dong, Dong-gu, Gwangju 501-759, Korea; E-Mails: sohuilove@nate.com (S.H.K.); luka10181215@empas.com (H.S.P.); jpark@chosun.ac.kr (J.P.)

**Keywords:** aged, influenza, vaccination, risk factors

## Abstract

The present study examined the rates and related factors for influenza vaccination among the elderly Korean population during the 2008/09 influenza seasons. We obtained data for 6,391 adults aged 65 years or older from Community Health Surveys conducted in 2009 and 2010 in 13 communities in the Honam region of Korea. A multiple logistic regression analysis was used to identify the factors associated with self-reported influenza vaccinations. In this elderly population, 81.7% reported to having received an influenza vaccination in the past year. The main contributing factors were older age, lower economic status, lower educational level, married, non-smoking, regular alcohol consumption, regular walking exercise, receiving a health check-up during the past two years, not stressed, and having comorbid conditions. The influenza vaccination coverage rate among elderly Koreans was relatively high, but improvements in vaccination rates are required.

## 1. Introduction

Influenza is a serious public health problem that is associated with elevated morbidity, mortality, and hospitalization, particularly in older adults (≥65 years old) and in those with cardiovascular disease, diabetes, chronic pulmonary disease, and other underlying medical disorders [1–6]. According to the World Health Organization (WHO), global influenza epidemics result in 3–5 million cases of severe illness annually, leading to 250,000–500,000 deaths. The majority of deaths associated with influenza in industrialized countries occur among citizens aged 65 or older [7].

Influenza vaccination has been demonstrated in numerous studies to be safe, efficient, and cost-effective. In the elderly population, the influenza vaccine is 50–60% effective in preventing hospitalization, and 80% effective in preventing death resulting from pneumonia and influenza [8–11]. The Korea Center for Disease Control and Prevention (KCDC) has recommended annual vaccinations to all citizens aged 65 years or older [12], and the vaccination is administered free of charge at public health centers in Korea [12]. From estimates of vaccine coverage among the elderly in the 2004/05 influenza season, 77.2–79.9% coverage was reported [2,13]. Although this range reached the national target which the KCDC had set a goal, that is to increase the vaccination rate in the priority group to reach at least 60% [12], coverage in specific elderly groups was inadequate. The factors identified as barriers to vaccination included gender, age, educational level, smoking, contact with the health care system, and comorbidities [1,2,14–17]. Identification of the associated factors in older adults is required to determine which segments of the elderly population should be targeted to improve the overall coverage of influenza vaccination programs [14,16–18].

To our knowledge, no previous study has examined the factors associated with influenza vaccination in Korea in a large representative sample of community-dwelling older adults. Based on data obtained from the 2009 and 2010 Community Health Surveys (CHSs) in 13 counties in the Honam region, this study investigated the influenza vaccination rate in the 2008/09 influenza seasons and identified factors associated with influenza vaccination among older adults.

## 2. Methods

### 2.1. Study Population and Data Collection

This study used pooled data collected from Community Health Surveys (CHSs) conducted by the Korea Center for Disease Control and Prevention (KCDC) and the public health centers of 13 communities in the Honam region of Korea in 2009 and 2010. Honam region is located in the south-west of Korea and consists of two provinces (North and South Jeolla) and one metropolitan city (Gwangju). The target area of this study is located in the central area of Honam region and consists of six urban communities and seven rural communities. The CHS has been conducted since 2008 by trained interviewers in annual face-to-face interviews across the nation and is designed to produce community-based comparable health statistics at the level of city (si), county (gun), and district (gu) units. A complex, stratified, multistage, probability-cluster sampling was used based on resident registration information. About 90 sampling units were randomly selected from the entire sampling units in each community, and with 5–8 households selected from each primary sampling unit, to yield an of average 900 subjects ≥19 years old from each community. Collected information includes socio-demographic characteristics, health behavior, chronic disease/injury, and quality of life/health services. The data used in this study were collected in September–November 2009 and August–October 2010 [19]. We limited the study population for this analysis to 7,089 elderly adults (≥65 years old). Among them, 6,391 elderly adults were finally identified as study subjects, after excluding respondents who did not know their influenza vaccination status (698 persons).

### 2.2. Study Variables

The influenza vaccination coverage was measured by a single question “During the past 12 months, have you been vaccinated against influenza?” The answer (yes/no) was used as the dependent variable. The purpose of this study was to identify factors associated with influenza vaccination among older adults in Korea. To perform this purpose, we tried to examine the possible factors associated with influenza vaccination which were selected through preceding studies. Therefore, selected variables were socio-economic variables (gender, age, educational level, monthly income, marital status, residency area), lifestyle and health-related variables (walking activity, smoking, alcohol consumption, stress, health check-ups, and co-morbidities).

The socio-economic variables were gender, age (65–74, ≥75 years), educational level (non-educated, primary school, higher than middle school), monthly income (≤1 million won, ≥1.01 million won), marital status (married, other), and residency area (urban, rural). The lifestyle and health-related variables were regular walking activity (at least 30 minutes at least three times a week), smoker (currently smoking), regular alcohol consumer (drinking alcohol more than twice a month), mental stress (yes/no), health check-ups during the past two years, and the presence of any co-morbidities that defined a recommendation for the influenza vaccination such as diabetes, stroke, myocardial infarction, angina, asthma, pulmonary tuberculosis, and hepatitis B.

### 2.3. Statistical Analysis

We conducted statistical analyses using the SAS 9.1 program (SAS Institute, Cary, NC, USA). A descriptive analysis of the variables was performed by gender, using the chi-square test and Fisher’s exact test when necessary. A bivariate analysis was performed between the vaccinated and unvaccinated patients for a comparison of all the independent variables. A multiple logistic regression analysis was used to identify the factors associated with the influenza vaccination with the inclusion of gender, age, and the variables that presented a p-value ≤0.10 in the bivariate analysis. We calculated the odds ratio estimates from the logistic regression model. A p-value ≤0.05 was considered statistically significant, and a 95% confidence interval (CI) was reported as appropriate.

### 2.4. Ethics Statement

The protocol of community health survey was reviewed and approved by the Institutional Review Board of Korean Centers for Disease Control and Prevention (2010-02-CON-22-P). Written informed consent was obtained from all participants in the community health surveys.

## 3. Results

Table 1 shows the socio-economic factors among the older adults by gender. Of the 6,391 older adults, 2,564 (40.1%) were men and 3,827 (59.9%) were women. The mean (standard deviation) age of the study subjects was 73.2 (6.1) years (men 72.8 (5.7) years, women 73.5 (6.4) years); the women were significantly older than the men. The men were more educated, had higher monthly incomes, a higher percentage were married (living with a partner or spouse), and a higher percentage of them lived in urban areas. Significant differences were observed between the genders in terms of socio-economic factors.

Table 2 shows the distribution of lifestyles and health-related factors among the older adults by gender. The percentage of current smokers for men was 25.1% and 3.8% for women (p < 0.0001). The percentage of regular alcohol consumers for men was 42.1% and 12.1% for women (p < 0.0001). Of the men, 45.2% participated in regular walking activity and 52.1% of women (p < 0.0001). In addition, significantly more men had received health check-ups during the prior two years than women. However, more women reported being stressed. The prevalence rate of co-morbidities of the men was higher than for women (33.4% men, 30.1% women, p < 0.0001).

Of the 6,391 older adults, the number of those vaccinated were 5,220 (81.7%) and the unvaccinated were 1,171 (18.3%). Table 3 summarizes the coverage rate of influenza vaccination according to socio-economic factors in older adults. Regarding the socio-economic factors, the coverage rate of influenza vaccinations was significantly different for education level (82.5% uneducated, 82.2% primary school and 78.2% higher than middle school, p < 0.05), for monthly income (82.9% under 1 million won, 79.1% above 1.01 million won, p < 0.05), for marital status (82.6% married, 80.1% other, p < 0.05), and for residency area (76.6% urban area, 82.6% rural area, p < 0.0001).

Table 4 was shown the coverage rate of influenza vaccination according to lifestyles and health-related factors in older adults. Non-smokers were significantly more likely to have received the influenza vaccination than smokers (82.4% nonsmokers and 76.7% smokers, p < 0.001), and the coverage rate of vaccination of the older adults who regularly walked was higher than that of those who did not walk regularly (84.5% and 78.8%, respectively, p < 0.0001). Also, older adults who did not report stress and who had received a health check-up during the past two years and who had co-morbid conditions (p < 0.05).

Results of multiple logistic regression analysis to determine factors associated with influenza vaccination are shown in Table 5. Among the older adults the variables that were independently and significantly associated with a higher likelihood of vaccinating the influenza were: (a) female (OR 1.21 95% CI 1.00–1.46), (b) monthly income ≤1 million won (OR 1.21 95% CI 1.03–1.41), (c) educational level; uneducated (OR 1.46 95% CI 1.17–1.82), primary school graduation (OR 1.27 95% CI 1.03–1.57), (d) being married (OR 1.26 95% CI 1.07–1.50), (e) regular alcohol consumer (OR 1.20 95% CI 1.00–1.42), (f) engaging in regular walking activity (OR 1.43 95% CI 1.25–1.65), (g) receiving a health check-up (OR 3.68 95% CI 3.19–4.24), and (h) having co-morbid conditions (OR 1.12 95% CI 1.06–1.40).

In addition, the variables independently and significantly associated with a lower likelihood of receiving the vaccine among the older adults were: (a) aged 65–74 years (OR 0.80, 95% CI 0.69–0.94), (b) smoker (OR 0.75, 95% CI 0.61–0.92), (c) being stressed (OR 0.77 95% CI 0.65–0.91).

## 4. Discussion

The rate of influenza vaccination coverage in the 2008/09 influenza seasons among the elderly respondents was 81.7%. This coverage shows improvement compared to other previous reports in Korea (77.2–79.7% in 2003/04) [2,13], and is relatively high compared to the coverage of the elderly in other counties such as Brazil (73%) [14], Canada (55.2%) [16], Germany (63.4%) [20], the US (70%) [21], Spain (63.7%) [15] and France (62.6%) [1], although the periods of data collection differed among these studies. The vaccination rates met the target set by the Korea Center for Disease Control and Prevention (KCDC) (>60%) [12], and also the WHO goal of 75% vaccination coverage within the elderly population by 2010 [22]. However, this rate is far from the international health objective for 2010 that aimed for at least 90% of the population aged 65 years or older to be vaccinated [23].

This study showed that older age increased the likelihood of vaccination, consistent with observations from other countries [14–16,24,25]. According to current Korean guidelines, the influenza vaccination is indicated for all persons aged 65 years and over [12]. If those at the younger end of this age group are less likely to be vaccinated, more must be done to ensure that the vaccination reaches the entire target population. The older adults who were vaccinated tended to have a lower level of education and received a lower monthly income, as previously identified [2,26]. This may be due to the government policy to administer the influenza vaccine free of charge to the vulnerable at public health centers [2]. However, previous reports have demonstrated that those with a higher educational and/or income level are more likely to receive a preventive health service such as a vaccination, possibly because they are more knowledgeable regarding the health services [16,24,25,27].

This study also demonstrates that married elderly individuals were more likely to be vaccinated. Previous studies also found more frequent use of preventative services in married couples because of improved social support, easier transportation, and greater likelihood of having previously established a regular source of health care [17,25,28]. The vaccination coverage of females and those living in rural areas was reported as significantly higher in a univariate analysis and other studies [2,14]. However, neither gender nor residence area retained a statistical significance in the multivariate analysis, suggesting that each was crude association influenced by other confounding factors [16].

Regular walking activity, currently non-smoking, regular alcohol consumption, and lack of stress were identified as associated factors with influenza vaccination. Other studies noted that these associations may have been confounded and are general health-protective behaviors; individuals exhibiting healthy lifestyle choices may be more likely to seek preventive health care and to visit their health care providers more regularly, thus providing more opportunity to be vaccinated [2,16]. These healthy lifestyle statistics did retain significance in our multivariate analysis. In theory, elderly smokers possess an increased incentive to receive the influenza vaccination, due to their increased likelihood of pulmonary complications. However, our data suggested that fewer smokers were vaccinated than non-smokers, consistent with reports from other studies [14,15].

Several previous studies found that individuals reporting a medical consultation in the previous year were more likely to be vaccinated as compared to those without a consultation [14,16,28]. Similarly, our results showed that those receiving a health check-up during the previous two years were more likely to be vaccinated [15–17,25,27,29]. Individuals who are more concerned about their health tend to visit doctors or nurses more regularly and are more likely to be aware of and use other health resources, such as vaccinations. The influence of co-morbidity as predictors of vaccination in the elderly has been reported [2,15–17,25,27,29]. Such patients are perceived by their health care providers as being at a higher risk of influenza infection and are thus more likely to be immunized [16].

The strength of this study lies in the fact that the CHS provides a large and unique data set of primarily community-dwelling older Koreans and is therefore potentially useful in the examination of health-related risk factors and demographics that may influence decisions to vaccinate. This study has several limitations. First, the information regarding influenza vaccination was collected from self-reports. The vaccination coverage rate could have been underestimated by the elderly respondents reporting their vaccination histories. However, self-reports of influenza vaccination status in older adults have been found to be highly sensitive and moderately specific when compared to medical records [30–32]. Second, the subjects in this study were representative of the population in Honam region and not necessarily the Korean population as a whole, meaning that extension of the inferences to the rest of Korea could be compromised. Therefore, studies on other region of Korea are needed to further confirm the associated factors. Third, the validity of the questions used to classify the subjects as having high-risk medical conditions have not been evaluated. Moreover, some conditions such as chronic renal disease, chronic liver disease, and cancer, which are associated with an increased risk of influenza complications, are not included in the CHS. Finally, the cross-sectional design of this study means that any association found between the vaccination coverage and variables does not involve a causal relationship.

## 5. Conclusions

Influenza vaccination coverage in Korea is relatively high; however, improvements are still required, particularly among the younger elderly those with no concomitant medical conditions, and smokers. More efforts should be paid to convince these unvaccinated groups of effectiveness of influenza vaccination, and to provide the exact information on influenza. This study may be useful in guiding efforts to increase the awareness among healthcare providers and develop systems to ensure that the vaccination levels are increased.

## Figures and Tables

**Table 1:** Distribution of socio-economic factors among the older adults in relation to gender.

Variables	Values	Gender		Total (n < 6,391)	p-value

		Male (n < 2,564)	Female (n < 3,827)		
Age groups (years)	68–74	1,714 (66.8) ^*^	2,441 (63.8)	4,155 (65.0)	0.0127
	75+	850 (33.2)	1,386 (36.2)	2,236 (35.0)	

Educational level	Uneducated	666 (26.0)	2,643 (69.1)	3,309 (51.8)	<0.0001
	Primary school	1,018 (39.7)	977 (25.5)	1,995 (31.2)	
	Higher than middle school	878 (34.3)	207 (5.4)	1,085 (17.0)	

Monthly income (won)	≤1 million	1,780 (72.7)	2,811 (77.7)	4,591 (75.7)	<0.0001
	≥1.01 million	668 (27.3)	807 (22.3)	1,475 (24.3)	

Marital status	Married	2,289 (89.3)	1,643 (42.9)	3,932 (61.5)	<0.0001
	Other	273 (10.7)	2,184 (57.1)	2,457 (38.5)	

Residency area	Urban	423 (16.5)	542 (14.2)	965 (15.1)	0.0117
	Rural	2,141 (83.5)	3,285 (85.8)	5,426 (84.9)	

Note: Data are presented as frequency (percentage).

**Table 2:** Distribution of lifestyles and health-related factors in relation to gender.

Variables		Gender		Total (n < 6,391)	p-value

		Male (n < 2,564)	Female (n < 3,827)		
Smoker	No	1,920 (74.9) ^*^	3,682 (96.2)	5,602 (87.6)	< 0.0001
	Yes	644 (25.1)	145 (3.8)	789 (12.4)	

Regular alcohol consumer	No	1,485 (57.9)	3,360 (87.9)	4,845 (75.9)	< 0.0001
	Yes	1,078 (42.1)	462 (12.1)	1,540 (24.1)	

Walking activity	Regular	1,160 (45.2)	1,990 (52.1)	3,150 (49.3)	< 0.0001
	Not regular	1,404 (54.8)	1,830 (47.9)	3,234 (50.7)	

Stressed	No	2,144 (83.9)	3,046 (80.0)	5,190 (81.6)	0.0001
	Yes	411 (16.1)	760 (20.0)	1,171 (18.4)	

Health check-up	No	588 (22.9)	1,029 (27.0)	1,617 (25.3)	0.0003
	Yes	1,975 (77.1)	2,788 (73.0)	4,763 (74.7)	

Co-morbidity	No	1,707 (66.6)	2,674 (69.9)	4,381 (68.5)	0.0059
	Yes	857 (33.4)	1,153 (30.1)	2,010 (31.5)	

Note: Data were presented as frequency (percentage).

**Table 3:** Influenza vaccination coverage rates according to socio-economic factors.

Variables		Vaccinated (n < 5,220)	Unvaccinated (n < 1,171)	p-value
Gender	Male	2,065 (80.5) ^*^	499 (16.5)	0.0582
	Female	3,155 (82.4)	672 (17.6)	

Age groups (years)	65–74	3,400 (81.8)	755 (18.2)	0.6939
	75+	1,820 (81.4)	416 (18.6)	

Educational level	Uneducated	2,730 (82.5)	579 (17.5)	0.0044
	Primary school	1,640 (82.2)	355 (17.8)	
	Higher than middle school	848 (78.2)	237 (21.8)	

Monthly income (won)	≤ 1 million	3,804 (82.9)	787 (17.1)	0.0013
	≥ 1.01 million	1,167 (79.1)	308 (20.9)	

Marital status	Married	3,249 (82.6)	683 (17.4)	0.0135
	Other	1,969 (80.1)	488 (19.9)	

Residency area	Urban	739 (76.6)	226 (23.4)	< 0.0001
	Rural	4,481 (82.6)	945 (17.4)	

Note: Data were presented as frequency (percentage).

**Table 4:** Influenza vaccination rates according to lifestyles and health-related factors.

Variables		Vaccinated (n < 5,220)	Unvaccinated (n < 1,171)	p-value
Smoker	No	4,615 (82.4) ^*^	987 (17.6)	0.0003
	Yes	605 (76.7)	184 (23.3)	

Regular alcohol consumer	No	3,934 (81.2)	911 (18.8)	0.0761
	Yes	1,282 (83.2)	258 (16.8)	

Walking activity	Regular	2,733 (84.5)	501 (15.5)	< 0.0001
	Not regular	2,481 (78.8)	669 (21.2)	

Stressed	No	4,289 (82.6)	901 (17.4)	0.0002
	Yes	913 (78.0)	258 (22.0)	

Health check-up	No	1,057 (65.4)	560 (34.6)	< 0.0001
	Yes	4,154 (87.2)	609 (12.8)	

Co-morbidity	No	3,543 (80.9)	838 (19.1)	0.0154
	Yes	1,677 (83.4)	333 (16.6)	

Note: Data were presented as frequency (percentage).

**Table 5:** The factors associated with influenza vaccination in the older adults.

Risk factors		Influenza vaccination (N < 6,022, Pseudo R^2^ < 0.11)		

		OR a	95% CI b	p-value
Gender (ref c < Male)	Female	1.21	1.00–1.46	0.046
Age (ref < ≥75 years)	65–74 years	0.80	0.69–0.94	0.006
Monthly income (ref < ≥1.01 million won)	≤1 million won	1.21	1.03–1.41	0.020
Educational level (ref < Higher than middle school)	Uneducated	1.46	1.17–1.82	0.003
	Primary school	1.27	1.03–1.57	0.038
Marital status (ref < Others)	Married	1.26	1.07–1.50	0.006
Residency area (ref < Urban)	Rural	1.16	0.96–1.41	0.122
Smoker (ref < No)	Yes	0.75	0.61–0.92	0.006
Regular alcohol consumer (ref < No)	Yes	1.20	1.00–1.42	0.045
Walking activity (ref < Not regular)	Regular	1.43	1.25–1.65	<0.001
Stressed (ref < No)	Yes	0.77	0.65–0.91	0.002
Health check–up (ref < No)	Yes	3.68	3.19–4.24	< 0.001
Co–morbidity (ref < No)	Yes	1.22	1.06–1.40	0.004

a Note: OR: Odds Ratio;

b 95% CI: Confidence Interval;

c ref: reference.
